# Age As Moderator of Emotional Stroop Task Performance in Posttraumatic Stress Disorder (PTSD)

**DOI:** 10.3389/fpsyg.2017.01614

**Published:** 2017-09-19

**Authors:** Maksymilian Bielecki, Agnieszka Popiel, Bogdan Zawadzki, Grzegorz Sedek

**Affiliations:** ^1^Department of Psychology, SWPS University of Social Sciences and Humanities Warsaw, Poland; ^2^The Robert B. Zajonc Institute for Social Studies, University of Warsaw Warsaw, Poland

**Keywords:** PTSD, emotional Stroop task, pictorial emotional Stroop task, attentional bias, aging

## Abstract

Emotional Stroop task (EST) has been extensively used to investigate attentional processes in posttraumatic stress disorder (PTSD). Even though aging significantly changes the dynamics of emotion-cognition interactions, very little is known about its role in shaping EST performance in PTSD patients. In the present study we tested a uniquely large sample of motor vehicle accident victims. Data of 194 participants (75.3% female; mean age = 36.64 years, *SD* = 12.3) were included in the analysis, out of which 136 (70.1%) were diagnosed with PTSD. Prior to the psychiatric assessment, participants completed the pictorial version of EST (neutral, positive, negative, and accidents photos were presented). Comparison of the PTSD and control groups revealed a specific increase in reaction times (RTs) related to the exposure of trauma-related material. At the same time, previously unreported, moderating effects of age were also discovered. Older participants, in contrast to the younger group, showed no increase in RTs and interference scores in trials where accident photos were presented. Our study points to the key role of age as a previously understudied factor modifying EST performance in PTSD patients.

## Introduction

Emotional Stroop task (EST) is one of the most important paradigms used to investigate attentional biases in a wide range of clinical populations including patients diagnosed with posttraumatic stress disorder (PTSD; Williams et al., 1996; McLeod, 2005). In the classic version of the EST, participants are asked to name the colors of sequentially presented words ignoring their meaning. Processing of the emotional content of the words imposes a cognitive load and leads to the delayed color naming. This delay, reflected in longer reaction times (RTs), is called emotional Stroop effect (ESE). In psychopathology of anxiety and stress-related disorders longer RTs are typically observed if stimuli are associated (emotionally or semantically) with participant’s perceived area of threat, and thus to a clinical diagnosis. Although some of the reported results are inconsistent (Kimble et al., 2009), current consensus clearly indicates that patients with PTSD performing the EST exhibit a specific increase in RTs when confronted with trauma-related stimuli (Cisler et al., 2011). The validity of the link between PTSD and emotional Stroop effect was further corroborated in studies showing its reduction after completing psychotherapy (Foa and Rauch, 2004) or special attentional training (Khanna et al., 2016).

There are also significant developments in our understanding of the cognitive mechanisms and neural underpinnings responsible for the emergence of the ESE, both in healthy (Ben-David et al., 2012; Ben-Haim et al., 2014) and clinical populations, including patients with anxiety disorders (for review, see: Cisler and Koster, 2010) and, more specifically, PTSD (Bruce et al., 2013; Shvil et al., 2013). There is also a growing body of knowledge concerning factors modifying the size of the ESE depending on the specific characteristic of the experimental procedure. For example, meta-analysis performed by Phaf and Kan (2007) showed that there was no detectable ESE if stimuli were shown “suboptimally,” i.e., using short presentation times and/or masking reducing the conscious perception of the presented material. ESE was also systematically larger in studies presenting blocks of homogenous stimuli when compared with experiments randomly mixing neutral and trauma-related words (see also Ben-Haim et al., 2014).

Despite the breadth of research using EST, existing data on effects of age are limited and partially contradictory. While some studies describe increase in ESE with age (Wurm et al., 2004), other suggest that older adults might be less affected by the emotional content of the stimuli – either in general (Lamonica et al., 2010) or in specific experimental settings (Ashley and Swick, 2009). Authors discussing these findings refer to many different non-exclusive explanations of the observed effects. Postulated mechanisms include age-related improvement in emotional regulation (Scheibe and Carstensen, 2010; Morgan and Scheibe, 2013), differences in amygdala responsiveness to threat signals (Lamonica et al., 2010), and age-related changes in working memory functioning (Ashley and Swick, 2009). The data concerning age effects in individuals exposed to traumatic experiences are even scanter. There are only two published papers (Wittekind et al., 2010, 2017) directly tackling that issue. Both tested a group of older adults traumatized during or just after the II World War. Obtained results were partially incoherent (no ESE in the earlier study) and hard to generalize due to the limited power, specificity of the tested sample (i.e., participants were diagnosed and tested more than 60 years after the traumatic events, the PTSD group included the subsyndromal cases), and limitations of the study design not including any younger control group.

Taking into account the multitude of potential theoretical explanations, diversity of the experimental procedures, and scarcity of the empirical material it is not yet possible to create a comprehensive theoretical model of the ESE that would allow to predict the moderating role of age in the PTSD population. At the same time, however, accurate identification of this role in clinical samples is essential for a number of reasons. Firstly, it would let to determine the limitations of the concurrent and predictive validity of the ESE when used in clinical setting. ESE is often proposed as an index that could support diagnosis in practical contexts, i.e., assessing changes induced by treatment (e.g., Gropalis et al., 2013) or predicting its success (Waters et al., 2003; Streeter et al., 2008). Secondly, it would allow to further verify the existing models of attentional biases of studied disorders. Finally, it might help explain some of the discrepancies in the literature on PTSD patients, as some publications (e.g., Kimble et al., 2009) question the very existence of the ESE in this population.

It is worth noticing that our current knowledge about PTSD does not allow us to address many other basic questions related to aging, including the fundamental one – whether or not age is significantly related to the risk of PTSD development following traumatic events. Current results provide mixed evidence (e.g., meta-analysis by Xue et al., 2015) and suggest that this relationship might be shaped by many variables, including social, economic, and cultural factors (e.g., Norris et al., 2002). Hence, looking at this issue from a broader perspective, a better understanding of the processes influencing participants’ behavior in relatively simple paradigms like EST might be also helpful in understanding the mechanisms of the disorder development and maintenance across the age-span.

According to our knowledge, our study is the first ever to directly address the question of the moderating effects of age on the ESE in the typical population of trauma-exposed subjects. Uniquely large sample with age ranging from 18 up to 69 years allowed us to use regression approach that has never been applied to study aging effects in PTSD. It is also the largest ever study conducted using the EST in a group homogenous regarding the type of stressor – all tested participants were motor vehicle accident (MVA) victims. At the same time, it is the only one using pictorial EST in this group, earlier reported effects were obtained using a verbal version of the task (e.g., Devineni et al., 2004). As outlined above, the ambiguity of the existing empirical material on aging effects on the ESE and limitations of data relevant to the PTSD patients determined the exploratory nature of our work. Drawing on the existing literature and concerning the sample size, we expected to observe significant effects related to the exposure of trauma-related material. We did not, however, form specific hypotheses addressing the moderating effects of age.

## Materials and Methods

### Study Design and Participants

This study was approved by institutional ethics committees at the University of Warsaw and the Military Institute of Aviation Medicine in Warsaw. It was conducted at the outpatient treatment center at the University of Warsaw during the screening phase of the program TRAKT. TRAKT (meaning “route” in Polish) was a clinical trial comparing the efficacy of different treatment modalities for PTSD (paroxetine vs. prolonged exposure vs. combined treatment) following MVA (for details: see Popiel et al., 2015).

#### Participants

The sample comprised of 202 participants who underwent clinical assessment at one of the diagnostic centers involved in the TRAKT project. Participants included in this sample completed a battery of computerized tests (including pictorial EST). Four participants were excluded on an a priori basis as they suffered from cognitive impairment due to traumatic brain injury resulting from MVA or an organic mental disorder. Further four were removed based on their low accuracy (details are provided in Data Analysis section below). The division into PTSD and non-PTSD control group was based on the results of the psychiatric diagnosis following criteria defined in DSM-IV-TR (American Psychiatric Association, 2000). Importantly, the two groups did not differ regarding their age, sex distribution, the proportion of participants who completed higher education, as well as the time that elapsed since the MVA (all details reported in **Table 1**). Informed consent had been obtained from all the participants prior to testing.

**Table 1:** Sample characteristics.

	PTSD (*n* = 136)	Control (*n* = 58)	Independent samples *t*-test		
	*M* ±*SD*	*M* ±*SD*	*t* (*df*)	*p*	Cohen’s *d*
Age (in years)	36.7 ± 12.1	36.5 ± 13.0	0.14 (101.2)	0.89	0.02
Time since MVA (in months)	20.6 ± 23.7	21.7 ± 29.2	-0.25 (90.4)	0.80	0.04
SCID-I symptoms	11.1 ± 2.56	3.29 ± 2.27	21.15 (120.6)	<0.001	3.23
	*n* (%)	*n* (%)	χ^2^(1)	*p*	Cramer’s *V*
**Sex:**					
Female	106 (69.0)	30 (77.9)			
Male	30 (31.0)	18 (22.1)	1.31	0.25	0.08
**Completed higher education:**					
No	59 (43.4)	27 (46.6)			
Yes	77 (56.6)	31 (53.4)	0.06	0.80	0.02

*MVA, motor vehicle accident; SCID-I symptoms, the sum of symptoms obtained in B, C, and D criteria during PTSD diagnosis based on Module F component of Structured Clinical Interview for DSM-IV Axis I Disorders.*

### Measures

#### Psychopathology

To establish the pre-treatment diagnoses entire Structured Clinical Interview for DSM-IV Axis I Disorders (SCID-I P/R; First et al., 1995) was administered to all the participants. PTSD was assessed using the SCID-I P/R Module F component (2008 revision). The SCID-I interviews were performed by five experienced psychiatrists. They were audio-recorded and 30% of them were rated by a different assessor for reliability, showing the good strength of agreement (κ = 0.70^∗^, 84,5% concordance of diagnoses). All these tools were adapted to the Polish language and have good psychometric properties (Zawadzki et al., 2015). Sample characteristics of both groups (**Table 1**) also include information about symptoms operationalized as their sum gathered in criteria B, C, and D during the interview establishing the PTSD diagnosis.

#### The Pictorial EST

In our choice of the EST characteristics, we aimed at maximizing the probability of evoking ESE while controlling for the potential confounding factors, which was of particular relevance given the socio-demographic heterogeneity of the sample. Drawing on the results of metaanalysis by Phaf and Kan (2007), we decided to present the stimuli in blocks and optimally, i.e., without any attempts to limit their conscious perception. The homogeneity of the stressor affecting our sample motivated the use of pictorial material that was relevant to the experiences of all the participants and, unlike words, should be processed similarly regardless of the level of education or reading proficiency.

Four categories of stimuli were used in the pictorial EST: positive, negative, neutral, and accident photos. All the photos were selected from the International Affective Picture system (IAPS; Lang et al., 1999), except accident photos, which were made available by Archives of Warsaw Police (11 photos) and supplemented by a single car accident photo from IAPS. All the accident photos had to depict crashed cars and accident scenes, but with no visible corps, injured bodies or blood, as we wanted the emotional interference to be specifically related to the memories of the accident, and not evoked by more general negative reactions (e.g., the view of mutilated bodies). Detailed list of the selected pictures from IAPS and their ratings might be found in **Appendix A**.

Each trial started with a black fixation cross (1000 ms), than a photo appeared for 600 ms, followed by a colored rectangle fully masking the picture and remaining on the screen until a response was made or 3000 ms elapsed. The stimuli were presented on a 15-inch color monitor using E-prime software (version 1.1.4.1; Schneider et al., 2002). Participants were asked to look at the screen and name the color (red, yellow, green, or blue) of the rectangle appearing right after the photo was presented. Responses were given by pressing one of four keys (z, x, n, and m on a standard keyboard). The instruction underlined that both speed and accuracy of reactions were of equal importance. As we expected older participants to show a general slowing of the reactions, the pictures and color patches masking them were presented sequentially so that all participants were exposed to the stimuli for the exact same amount of time, independent of their response speed. After 12 practice trials, participants solved four blocks of trials. In every block all photos from a single stimulus category were presented, each appeared four times coupled once with each of the colors, forming 48 trials per block. At the end of each block participants could take a short break, before pressing the spacebar to start the next set of trials. Blocks and trial order was fully randomized across participants.

### Procedure

All participants were tested individually. The pictorial EST was solved as the last part of a larger battery of computerized tests. Notably, none of the tasks preceding the EST employed pictorial material related to MVAs. Testing session lasted approximately 50 min and was performed shortly before the diagnostic interview.

### Data Analysis

Results from four participants with distinctively low accuracy levels in the pictorial EST were removed prior to the analysis (in removed cases mean accuracy in at least one of the conditions was below 0.5 threshold, for the cases remaining in the analysis all accuracies were above 0.70). Demographic and basic clinical characteristics of the remaining 194 subjects are reported in **Table 1**. Analyses were conducted using three groups of indicators: (a) accuracy means, (b) RT means, and (c) difference scores computed by subtracting mean of RTs in neutral trials from mean scores computed for each of the remaining conditions (positive, negative, and accident photos). All incorrect responses and first trial in each block were removed from RT analysis. RTs shorter than 300 ms and longer than 3 SDs above the subject’s block mean were also discarded (RT trimming removed 0.79% of all observations). In the first step of our analysis, we focused on the influence of PTSD diagnosis on all the dependent variables. In the second step – on the moderating effects of age.

## Results

### Effects Related to PTSD Diagnosis

#### Accuracy

Mixed-design ANOVA was used to compare mean accuracies with Diagnosis (PTSD group vs. no PTSD) as between-subject factor and Photo Type (Neutral, Positive, Negative, Accident) as the within-subject variable. Results revealed a significant main effect of the Photo Type, *F*(2.1,398.1) = 11.79, *p* < 0.001, $\eta_{p}^{2}$ = 0.06. Due to the violation of the sphericity assumption effects including Photo Type in all analyses are reported with Greenhouse-Geisser correction). Tukey *post hoc* tests indicated that performance in Accident condition (*M* = 96.3%, *SD* = 5.59%) was significantly impaired when compared with Positive (*M* = 98.2%, *SD* = 2.37%, *p* < 0.001) and Neutral (*M* = 98.0%, *SD* = 3.00%, *p* < 0.001) trials and did not differ significantly when contrasted with trials presenting Negative material (*M* = 97.0%, *SD* = 4.36%, *p* = 0.133). Negative trials yielded lower accuracies when compared to trials with Positive pictures (*p* = 0.009). The remaining *post hoc* comparisons were non-significant. No effects related to the PTSD diagnosis were present, as both the main effect and the Diagnosis × Photo Type interaction were non-significant (*F*s < 1).

#### RT Mean Scores

**FIGURE 1:**
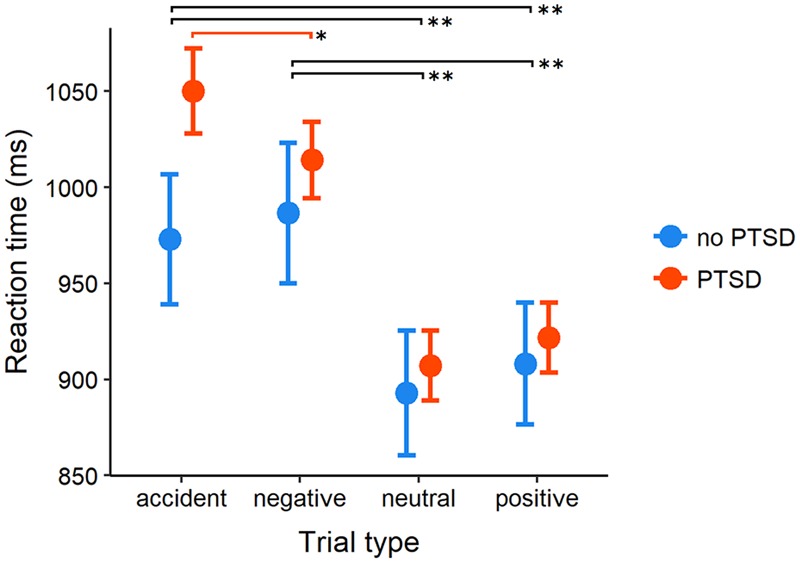
Mean reaction times (RT) (in milliseconds) in posttraumatic stress disorder (PTSD) and control group across four types of stimuli in the pictorial Emotional Stroop Task. Error bars indicate 95% between-subject confidence intervals of the mean. *p*-value for between-group comparison is reported for accident photos, in all the remaining conditions *p*-values were above 0.5. Black lines indicate significant between-condition *post hoc* comparisons present in both groups. Red line indicates the comparison significant within the PTSD group only. ^∗∗^*p* < 0.002, ^∗^*p* < 0.05.

In RT analysis we used the same ANOVA design as previously. As expected, the main effect of Photo Type [*F*(2.6,495.9) = 45.05, *p* < 0.001, $\eta_{p}^{2}$ = 0.19] was accompanied by the two-way interaction with Diagnosis, *F*(2.6,495.9) = 3.08, *p* = 0.034, $\eta_{p}^{2}$ = 0.02 (**Figure 1**). The main effect of Diagnosis was non-significant (*F* < 1). To disentangle the interaction effect, we performed analyses within groups. The main effect of Photo Type was present both in PTSD [*F*(2.4,327.5) = 50.66, *p* < 0.001, $\eta_{p}^{2}$ = 0.27] and no PTSD participants [*F*(2.8,158.7) = 13.83, *p* < 0.001, $\eta_{p}^{2}$ = 0.20], however, the pattern of results was markedly different. In both groups, Tukey’s *post hoc* tests showed that RTs in neutral and positive trials were shorter than in conditions were accident and negative photos were presented (all *p*s ≤ 0.002). Direct comparison of accident and negative trials showed, however, that significant increase in RTs related to the presentation of trauma-related material characterized only participants with PTSD (*p* = 0.049, *d* = 0.23) with no equivalent result in the control group (*p* = 0.867, *d* = -0.09). Between-group comparison of RTs in Accident trials did not reach statistical significance, *t*(107.91) = 1.91, *p* = 0.059, *d* = 0.30. (This and all the following independent samples *t*-test results are reported with Welch correction). Between subject comparisons in the remaining three types of trials were also non-significant (*p*s > 0.5, |*d|*≤ 0.11).

#### RT Difference Scores

**FIGURE 2:**
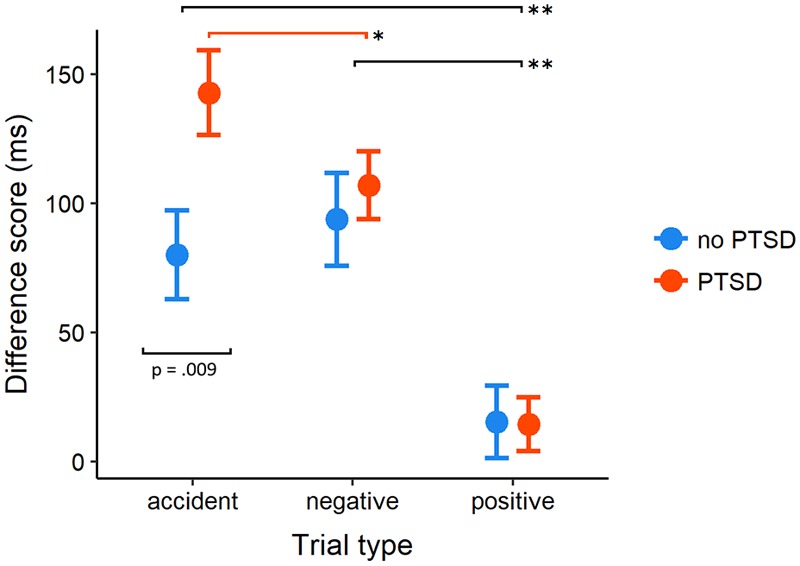
Mean interference score of RTs (in milliseconds) in PTSD and control group computed as the difference between mean RT in negative, positive, and accident vs. neutral trials in the pictorial emotional Stroop task. Error bars indicate 95% between-subject confidence intervals of the mean. *p*-value for between-group comparison is reported for accident photos, in all remaining conditions *p*-values were above 0.5. Black lines indicate significant between-condition *post hoc* comparisons present in both groups. Red line indicates the comparison significant within the PTSD group only. ^∗∗^*p* < 0.002, ^∗^*p* < 0.05.

Analysis of difference scores was based on 2 × 3 mixed-design ANOVA with two levels of Diagnosis and three levels of Photo Type (Positive, Negative, and Accident conditions, **Figure 2**). Results revealed that the main effects of Photo Type [*F*(1.9,369.2) = 35.81, *p* < 0.001, $\eta_{p}^{2}$ = 0.16] was accompanied by a significant interaction, *F*(1.9,369.2) = 3.58, *p* = 0.031, $\eta_{p}^{2}$ = 0.02. The main effect of Diagnosis was not significant, *F*(1,192) = 1.85, *p* = 0.175, $\eta_{p}^{2}$ = 0.01]. Separate analyses in both groups showed that the Photo Type influenced interference scores in both groups, however, the effect in the PTSD group was more pronounced [*F*(1.84,247.7) = 43.50, *p* < 0.001, $\eta_{p}^{2}$ = 0.24] than in non-PTSD subjects, *F*(1.96,112.0) = 9.95, *p* < 0.001, $\eta_{p}^{2}$ = 0.15. The pattern of within-group pairwise comparisons matched exactly the one observed in the previous analysis for the Positive, Negative, and Accident conditions (for each subject the same value was subtracted to compute the interference scores, so relative results did not change). The difference scores allowed to reveal a significant between-group effect. When subjects diagnosed with PTSD were contrasted with control participants, their average scores in Accident trials were significantly higher [*t*(164.5) = 2.64, *p* = 0.009, *d* = 0.35], whereas virtually no effects were present in the interference scores in negative and positive trials (*p*s > 0.5, |*d|*≤ 0.09).

### Moderating Effects of Ages

**FIGURE 3:**
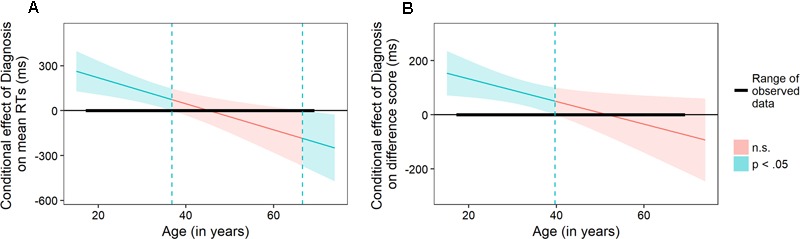
Johnson–Neyman regions of significance for the conditional effect of Diagnosis on mean RT in accident photo trials **(A)** and mean interference scores (computed as the difference between accident and neutral photos conditions, **B**) in pictorial emotional Stroop task. Color bands depict 95% confidence intervals – blue regions indicate significant and red – non-significant conditional effects of Diagnosis at given age.

Regression analysis was conducted to address the crucial question of the moderating role of age on the relationship between the PTSD diagnosis and ESE. Separate regression models were fitted for two key indicators: mean RTs in which trauma-related photos were presented and interference scores obtained for the same trials. Both models were estimated using HC3 heteroskedasticity-consistent standard error estimators (*sandwich* package implementation, Zeileis, 2006) using R statistical environment (R Core Team, 2016). Both analyses revealed the significant interaction of Age and Diagnosis on ESE (summaries of both models are presented in **Table 2**). To elucidate the moderating effects of age we used the Johnson–Neyman procedure (Hayes and Matthes, 2009). This technique allows to estimate the size of the effect related to the independent variable over the full spectrum of moderator values. Using Johnson–Neyman approach, we determined the “regions of significance,” i.e., age ranges for which the PTSD diagnosis modifies significantly the performance. **Figure 3A** shows that estimated value of the conditional influence of PTSD diagnosis on mean RTs decreased systematically becoming non-significant for participants above the age of 36.8 and even significantly negative for participants above the age of 66.6. This latter value is close to the upper range of the age values observed in our sample, as the oldest tested participant was 69. Similar results could be observed for difference scores (**Figure 3B**), however, in this analysis region of significance reached up to the value of 39.7. It is important to underline that obtained cutoff scores are determined not only by the slope of the conditional effect but also by the breadth of the confidence intervals. Estimated conditional effects of PTSD diagnosis reached zero (points where the conditional effect crosses the horizontal axis) at higher age values: 45.3 and 51.7 years of age.

**Table 2:** Summary of regression models describing the association between PTSD diagnosis and pictorial emotional Stroop task performance including age as a moderating variable.

	DV: Mean reactions times				DV: Difference scores			
	*B*	*SE*	*t*	*p*	*B*	*SE*	*t*	*p*
Intercept	615.5	84.5			97.2	52.1		
Age	9.8	2.2	4.56	<0.001	-0.5	1.5	-0.31	0.756
Diagnosis	393.3	106.4	3.70	<0.001	216.4	67.1	3.23	0.001
Age × Diagnosis	-8.7	2.8	-3.10	0.002	-4.2	1.9	-2.25	0.025

*DV, dependent variable; SE, standard error of the estimate. Models were fitted for two dependent variables quantifying reactions to trauma-related photos: mean reaction times (RT) and difference scores (after subtracting average RTs for neutral material). Diagnosis encoded group membership as binary variable (control group = 0, and PTSD group = 1).*

**Table 3:** List of photos from the International Affective Picture System used as stimuli in pictorial version of emotional Stroop task.

	Identification numbers of selected photos		
	Negative	Neutral	Positive
	2800	1670	1440
	2900	2200	1710
	3230	2215	1750
	3300	2372	1920
	6260	2749	2040
	6313	5534	2150
	6350	7002	2165
	9000	7009	2340
	9001	7035	2540
	9040	7050	2550
	9041	7150	5760
	9570	7705	7330

	*M* (*SD*)	*M* (*SD*)	*M* (*SD*)

Valence ratings	2.27 (0.50)	5.07 (0.59)	7.97 (0.25)
Arousal ratings	5.50 (1.17)	3.12 (0.45)	4.54 (0.59)

*Ratings and numbering based on Lang et al. (1999).*

Potentially, the effects of age could also be confounded by not taking into account the time elapsed between the MVA and the diagnosis. That was, however, not the case in our study. The amount of time that passed since the accident was not related to any of the variables in models reported above – there were no group differences (see **Table 1**) and correlations with age as well as both RT measures were non-significant and negligible (all |*rho*|*<* 0.1 and *p* ≥ 0.2). Same correlations were also estimated separately within PTSD and control group, again not yielding any significant results. There was also no significant correlation between participants’ age and the number of symptoms, as measured by SCID-I.

Additionally, we checked if the moderating effects reported above remained significant after adding participant sex or level of education as covariates. As none of the additional effects were significant, detailed descriptions of the results will be skipped for the sake of brevity.

## Discussion

According to our knowledge, this is one of the few studies focusing on the influence of age on ESE and the first one examining such a large sample of trauma victims. Using regression approach we showed that the interference effect observed in reaction to trauma-related photos in PTSD patients systematically decreased with subject’s age. Importantly, moderating effects of age were present both in RTs and interference measures and could not be explained by differences in other patient characteristics (sex, the level of education, time since MVA).

It is worth to notice that the lack of ESE in the older group of subjects diagnosed with PTSD is coherent with two different lines of interpretation. It might either be related to the diminished sensitivity to accident photos in older subjects with PTSD or relative increase in the emotional interference in the control group. Given the scant literature on the aging effects on ESE, deciding between these two interpretations is a non-trivial task.

First, we focus on the explanations supporting the hypothesis of reduced interference in the PTSD group. The mechanism responsible for diminished ESE could be a specific case of a broader phenomenon observed in older healthy subjects. In the general population, older people are characterized by a more effective emotional regulation (Scheibe and Carstensen, 2010; Hay and Diehl, 2011) and their attentional processes are biased toward positive stimuli (Reed et al., 2014). Furthermore, many studies indicate that in this group some components of the physiological reactions evoked by negative material are markedly different. Such effects were reported in studies using cardiovascular (e.g., lower heart rate reactivity, Uchino et al., 2010), breathing (e.g., no effects of arousal on inspiratory time, Gomez et al., 2016) and neuroimaging data (e.g., investigating the hypothesis of fronto-amygdalar age-related differences in emotion, St. Jacques et al., 2009). Potentially, age-related dampening of various components of physiological reactions (either peripheral or central) evoked by emotional material could also help explain the reduction of interference observed in older PTSD patients.

However, even if we ignore all the contradicting results and moderators of the effects listed above (e.g., Wurm et al., 2004; Uchino et al., 2010), it is important to notice that these explanations point toward more successful regulatory processes allowing older subjects to achieve positive emotional balance. That optimistic picture, described in the title of a review paper by Mather (2012) as “*The emotion paradox in the aging brain*,” does not seem to be adequate when juxtaposed with the core symptoms of PTSD (Shepherd and Wild, 2014). Intense anxiety, reexperiencing, and intrusive thoughts are the exact opposite of superior emotional control. Attributing more effective regulation to the older PTSD group is even less convincing, in the light of recent neuroimaging data. Effective coping with trauma-related stimuli correlates with increased top-down attentional control (White et al., 2015), whereas the behavioral data show that PTSD patients are characterized by lower levels of executive control both before and after the occurrence the traumatic stressor (Buckley, 2000; Aupperle et al., 2012). In sum, in our opinion, the age-related improvement in emotional regulation cannot account for the pattern of results observed in our study.

A more convincing explanation of the reduced ESE refers to the situational factors which are known to moderate the size of emotional interference and diversely influence participants of different ages. ESE is diminished if participants expect a confrontation with a significant real environmental stressor (Helfinstein et al., 2008). Similar effects were also observed in PTSD (Constans et al., 2004). Concerning the fact that the age range in our sample exceeded 50 years, it is likely that cohort effects and natural correlates of the aging might have shaped subjects reactions to the experimental task. In particular, both computerized testing situation and prospects of getting a psychiatric evaluation might have been perceived as stronger stressors by older subjects and, in consequence, affect the ESE in our study. Regrettably, since no measures of stress or emotional state were used during this part of the diagnostic process, we could not empirically verify this hypothesis. Future studies would certainly benefit from including momentary measurements of mood and stress levels as additional controlled variables.

The second line of reasoning explaining observed results refers to the hypothesis that aging makes all study participants – including the control group – more susceptible to the emotional load imposed by accident photos. It is important to underline that our sample consisted of people who decided to seek help and undergo a psychiatric evaluation following an MVA (for a detailed description of the treatment program see: Popiel et al., 2015). Hence, for every subject in our study accident pictures related to a significant, emotionally charged personal experience. Older adults are characterized not only by a general depletion of cognitive resources (e.g., Verhaeghen and Salthouse, 1997), but also by more specific changes in their emotional reactions including a gradual increase in aversive activation and the strength of arousal evoked by displeasure (Keil and Freund, 2009). The modulating effects of age on cognition–emotion interaction also translate into systematic changes in the perception of emotionally charged stimuli. As shown by Grühn and Scheibe (2008) older participants’ ratings of negative pictures from IAPS database are more extreme in terms of both affect and arousal. As mentioned before, in many contexts older people outperform younger groups in tasks requiring emotional regulation. This advantage, however, is not ubiquitous and several studies indicate that effects of superior emotional control might dissipate or even be reversed in certain conditions. For example, the positivity effects seem to rely on the cognitive-control-based allocation of attention. Therefore, the tendency to avoid negative stimuli is reversed if control processes are disrupted by the cognitive load imposed by a secondary task (Knight et al., 2007). Results obtained by Tomaszczyk et al. (2008) suggest that the positivity bias in memory recall might be also significantly reduced if participants are presented with pictures of high personal relevance (as it was the case in our study).

This line of reasoning might seem incompatible with the existing data concerning the ESE in elderly subjects’. It is known that ESE is preserved in older age in many groups including, for example, subjects who are depressed (Dudley et al., 2002; Epp et al., 2012) or habitually worrying (Price et al., 2012). Older participants are generally slower, but the emotional interference effects might be reliably observed. We should keep in mind, however, that these results are obtained in research involving healthy controls, whereas in our study, all participants were affected by a significant stressor – MVA. Even though the intensity of symptoms was much higher in PTSD, the control subjects were not fully asymptomatic (see: **Table 1**., SCID-I symptoms’ measure). At the same time, current knowledge about the ESE phenomenon (Phaf and Kan, 2007) suggests that it does not rely on the same regulatory mechanisms as the ones which are known to be more effective in older adults. For example, it would be hard to explain how ESE could be reduced by consciously changing the strategy of attention allocation (as it was the case in studies mentioned above).

All these results suggest that the older MVA victims, even if not presenting full spectrum of PTSD symptoms, might face more problems with effective functioning in accident trials when compared with younger subjects, and this could lead to the reduction of the between-group differences in ESE in our study. Given the complexity and variety of theoretical contexts, the in-depth analysis of the mediators responsible for diminished ESE in older participants remains as a task to be explored. The list of candidate mechanisms outlined above is certainly non-exhaustive, and it is also theoretically plausible that some of them might work in parallel. Moreover, our study design did not allow us to systematically investigate the time dynamics of the ESE, which is a topic of important theoretical implications (see e.g., Kunde and Mauer, 2008; Ben-Haim et al., 2014). In our procedure, each of the photos was presented four times in a fully randomized manner, hence changes in the ESE due to the stimulus repetition could not be disentangled from other important factors (gradual mood changes, habituation, etc.). More in-depth analysis of this aspect of participants’ performance is a task of the future.

Due to its exploratory character, our study certainly requires further validation. If such efforts were successful, the conclusions would bear important implications for the understanding of ESE from a measurement-oriented perspective. Tasks used in cognitive psychopathology research are often perceived as if their validity across socio-demographic dimensions were warranted. Our study might be treated as a reminder, that psychometric properties of any tool should be re-established whenever it is used beyond the typically tested population. That conclusion becomes even more relevant given the rapid aging of modern societies (United Nations, 2015) and the breadth of ESE usage, including studies on the effectiveness of therapeutic interventions (e.g., El Khoury-Malhame Myriam et al., 2011), neural underpinnings of various disorders (e.g., Herd et al., 2006; Cisler et al., 2011; Shvil et al., 2013) or relapse prediction in addicts (e.g., Waters et al., 2003; Streeter et al., 2008).

To summarize, our results point toward the importance of age as a variable moderating the presence of ESE in PTSD patients when contrasted with trauma-exposed controls. Concerning the exploratory nature of the study, reported results certainly require independent replication also allowing to determine to what extent our findings generalize to other clinical populations and different versions of EST, in particular – ones based on non-pictorial stimuli. It is important to underline, however, that current knowledge about the determinants of ESE, the emergence of the expected interference effects in younger subjects, and exceptionally large clinical sample add credibility to the postulated age-related decline of ESE.

## Ethics Statement

This study was carried out in accordance with the recommendations of the institutional ethics committees at the University of Warsaw and the Military Institute of Aviation Medicine in Warsaw. All subjects gave written informed consent in accordance with the Declaration of Helsinki. The protocol was approved by the institutional ethics committees at the University of Warsaw and the Military Institute of Aviation Medicine in Warsaw.

## Author Contributions

All the authors had contribution to the conception of the work and participated in the writing of the manuscript. MB was the lead author and primary writer. MB and GS contributed to the experimental procedure design. Data collection and psychiatric diagnoses were performed under the supervision of AP and BZ. MB performed data analysis. All authors approved the final version of the manuscript.

## Conflict of Interest Statement

The authors declare that the research was conducted in the absence of any commercial or financial relationships that could be construed as a potential conflict of interest.

## Appendix

Three categories of photos used in the pictorial version of the emotional Stroop task (negative, neutral, and positive) were selected from International Affective Picture System (Lang et al., 1999). **Table 3**. provides a complete list of picture numbers and descriptive statistics summarizing affective properties of each category of stimuli. Valence, and arousal ratings and photos’ numbers are based on Lang et al. (1999) publication (norms for both sexes). Selected pictures were horizontally oriented, did not depict any cars or elements that could be directly associated with accidents. In particular, negative pictures did not depict mutilated bodies or blood. Picture ratings are expressed on a 1 to 9 scale with 5 being the neutral middle-point of the valence scale.
